# Mahamari Plague: Rats, Colonial Medicine and Indigenous Knowledge in Kumaon and Garhwal, India

**DOI:** 10.1080/01459740.2022.2058397

**Published:** 2022-05-17

**Authors:** Christos Lynteris

**Affiliations:** Department of Social Anthropology, University of St Andrews, St Andrews, UK

**Keywords:** Colonialism, India, indigenous knowledge, Mahamari, plague, rats

## Abstract

Colonial approaches to animal and zoonotic diseases are often scrutinized in terms of their recognition or dismissal of indigenous knowledge. In this article I examine British colonial approaches to “Mahamari plague” in mid-nineteenth century Kumaon and Garhwal, in the Indian Himalayas. Discussing two key colonial medical expeditions in the region, I argue that the eventual recognition of the validity of Kumaoni and Garhwali knowledge of Mahamari and its relation to rats intensified intrusive colonial intervention on indigenous lifeways. I examine this neglected impact of the colonial recognition of indigenous knowledge and urge for approaches that place more emphasis on the practical impact of colonial epistemologies.

The ways in which colonial authorities approached and interacted with indigenous knowledge in different imperial contexts has long been the subject of systematic historical and anthropological examination. Reflecting a broader trend in the history and anthropology of medicine, in recent years a number of works have focused on recovering indigenous knowledge of animal and zoonotic diseases that preceded or competed with colonial medicine, demonstrating how, in many cases, the former also significantly informed or modified the latter (Beinert et al. 2009; Brown 2008a). In a key work on the subject, Clapperton Chakanetsa Mavhunga (2018) has shown how the control of trypanosomiasis in colonial Zimbabwe drew on indigenous knowledge about the tsetse fly as a vector of the disease. Working with Kapil Raj’s position (Raj 2007:13) that the colonies were “not a space for the simple application of European knowledge, nor a vast site for the collection of diverse information to be processed in the metropolis,” but rather “active, although unequal, participant[s] in an emerging world order of knowledge,” other works have mounted sustained critiques of the colonial-indigenous knowledge dichotomy. Rohan Deb Roy (2018:151) has noted that, when solicited by colonial authorities, indigenous opinions about diseases and epidemics “often reflected the understandings and biases of the colonial state.” Going beyond already established framings of the relation between colonial and indigenous medicine as one of “codification” and “valorization” (Tilley 2011), new studies focused on colonial and postcolonial East and southern Africa have shown that indigenous knowledge of animal diseases has been in “interplay” with biomedicine (Brown et al. 2013:324) in dynamic ways that defy dichotomization: “African veterinary knowledge was neither static nor isolated, but rather accretive, drawing on Western knowledge at times, and both were syncretic and shifting” (Mwatwara and Swart 2015:113). At the same time, I (2016) have demonstrated that indigenous knowledge of plague and its animal reservoir (Siberian marmots) among Mongols and Buryats in Qing China and the Russian Empire was in fact a late-nineteenth-century colonial, medical materialist construct, which curtailed the autonomy of indigenous modes of knowledge, reducing these from complex pathways of interspecies interconstitution to a fossilized, protoscientific repository of information about a specific, biomedically defined disease.

In this article, I return to a classic locus of studies of colonial-indigenous interaction, India, so as to examine a neglected case of colonial encounter and interaction with indigenous knowledge, which took place in the “scientific locality” (Chambers and Gillespie 2000:228) of a series of outbreaks of a disease known as “Mahamari” or the “Mahamari plague” in mid-nineteenth century Kumaon and Garhwal (the Himalayan areas today belonging to the Indian State of Uttarakhand).^1^ I will examine how colonial doctors evaluated and incorporated indigenous knowledge of and approaches to Mahamari as a disease that first struck rats and then humans in their examination of the nature of the disease, its identity as true plague, its causes, and the ways to contain and prevent it before the emergence of bacteriology and the microbiological identification of plague and rats as its zoonotic hosts.

In his contribution to *The New Cambridge History of India* (“Science, Technology and Medicine in Colonial India”), David Arnold (2000:68) argued that if up to the late 1830s, “Early British attitudes to what was known as Ayurvedic, Unani and even folk medicine were often tolerant and even appreciative,” the rise of medical topography and a focus on the environment, which would eventually lead at the end of the century to the emergence of tropical medicine, fostered a categorical dismissal of indigenous knowledge. Arnold has qualified this periodization with examples in the second half of the nineteenth century where “a process of partial interaction and assimilation” between colonial medicine and indigenous knowledge “did occur” (Arnold 2000:70). More recently, historians (e.g. Brimnes 2013; Harrison 2001; Hochmuth 2006; Mukharji 2009) have come to further trouble this periodization and show that British colonial approaches to medical and public health questions and indigenous knowledge interacted in complex ways, which differed significantly “not only according to time and place but also according to administrative context” (Brimnes 2013:77).

The case examined in this article follows a reverse order to that suggested by Arnold: a movement from a colonial dismissal of indigenous knowledge of Mahamari to its recognition. Rather than, however, reading this as evidence against the periodization framework prevalent in historical studies of colonial medicine in India, I would like to argue that the case of mid-nineteenth century Mahamari (that is, Mahamari before the bacteriological identification of plague and the start of the third plague pandemic) poses a more fundamental challenge to the way in which we approach colonial medical encounters and interactions with indigenous knowledge. A common ground or analytical baseline of historical and anthropological works on colonial medicine in India and elsewhere has been a focus on whether or not colonial officers and doctors recognized or dismissed the validity and value of indigenous knowledge. However, this focus overlooks the fact that in some cases, such as the one examined in this article, the recognition of indigenous knowledge on the part of colonial doctors led to a much more totalizing pathologization of indigenous lifeways and forceful “intrusive intervention” (Mooney 2015) on the ground than the dismissal of this knowledge previously had. In this article, I aim to examine this neglected impact of recognizing indigenous knowledge, through a comparison of two key colonial medical framings of Kumaoni and Garhwali approaches to Mahamari before the advent of bacteriology (1870s), the third plague pandemic (1894–1959), and the bacteriological identification of plague (1894) and of rats and their fleas as the disease’s hosts and vectors (1898).^2^

This article relies on an anthropological interrogation of the colonial archive; in other words, it examines archives, as Ann Laura Stoler (2008) has argued, “along their grain” as sites of contested knowledge, by taking their producers ethnographically seriously and investigating the “epistemic anxieties” and the “colonial common sense” in place. Approaching colonial archives “not as sites of knowledge retrieval but of knowledge production, as monuments of states as well as sites of state ethnography” (Stoler 2002:90), I will investigate how the interpretation of data about indigenous knowledge – data classed here as “ethnographic” insofar as they formed part of a much broader project and network of colonial information gathering about the lifeways of colonized peoples in India (Bayly 1996) – impacted epidemiological reasoning and colonial public health policy in mid-nineteenth-century India.

I will show how, in the course of the first colonial medical investigation on Mahamari (by the Superintending Surgeon of the Meerut Circle, Dr C. Renny – 1849), indigenous knowledge of rats dying before humans was overlooked and indigenous response to both the mass death of rats (ratfalls) and human deaths (fleeing to the hills) was framed as superstitious and pathogenic, due to it resulting in supposedly pestilential corpses being abandoned in the evacuated villages. I will then discuss how this approach to indigenous knowledge was challenged in the second investigation of the disease (by the colonial Medical Officers Francis and Pearson – 1852-53), which established the validity of indigenous observations of ratfalls preceding human outbreaks and of the strategy of fleeing infected villages, only however to interpret the death of rats in such as way as to put epidemic blame on Kumaoni and Garhwali housing. I will argue that this marked a significant shift in epidemiological reasoning and epidemic blame, which by taking indigenous knowledge and response seriously ended up pathologizing no longer an isolated “superstition,” i.e. a set of supposedly erroneous ideas or beliefs, but the very infrastructure of Kumaoni and Garhwali lifeways – the material conditions and arrangements of indigenous life. Finally, I will show how, a decade later (1861), in his capacity as Sanitary Officer of Garhwal, Pearson would add a third, racialized enclosure of indigenous knowledge of Mahamari by accusing Garhwalis of failing to act upon their knowledge of ratfalls preceding human cases and flee their villages.^3^

## Renny’s expedition

Following the Anglo-Nepalese War of 1814–16 and the Treaty of Segauli (1816) between the British East India Company and the King of Nepal, the eastern half of what before its annexation by the Gorkhas was the kingdom of Garhwal came under British rule under the administrative name of the Non-Regulation Province of British Kumaon and Garhwal. In 1836 the region was incorporated into the North-Western Provinces, and in 1839 it was divided into the mainly Hindu districts of Kumaon and British Garhwal, covering an area of 11,038 square miles, with the location of the majority of settlements located at an altitude of 900 to 1800 meters (Bergmann 2016; Hutcheson 1895; Mittal 1986).^4^ On December 22, 1849, The Senior Assistant Commissioner of Garhwal, John Strachey, informed the fifth Commissioner of Kumaon, John Hallet Batten, that an outbreak of a disease known as “Mahamurree” (later spelled *Mahamari*) had broken out with great force in the district of Chaprakot (Stratchey to Batten, December 22, 1849; BL, IOR/V/27/68/233:441–442).^5^ Having raged for four months, Mahamari was reported to have decimated the villages of Marora and Dadoli, killing a quarter of their population. Fearing that the disease was plague, Strachey urged Batten to order an inquiry into the epidemic. From the very start of the outbreak, ethnographic information (broadly defined as data about indigenous lifeways) was of importance in evaluating the situation, and deciding the next steps in both research and public health policy. News that the local population considered this to be a disease so contagious that they abandoned their sick relatives and fled for the surrounding forests and hills alarmed the Commissioner, who feared this could be the start of a spread of the disease from its hitherto isolated abodes in the Himalayan highlands: “The plague is undoubtedly coming lower and lower every year” (Batten to Thornton, January 1, 1850; BL, IOR/V/27/68/233:443).

This was not the first time that the colonial government in Kumaon and Garhwal had heard of Mahamari. Not to be confused with the use of the same term for cholera outbreaks in other parts of India, the “Mahamari plague” had first been recorded in 1823 in the Himalayan pilgrimage town of Kedarnath, when reportedly, “the late Rawul of the temple of Kedarnath, in the performance of the religious ceremony called ‘hom,’ deviated from the rules prescribed by the shasters and in consequence died, together with the Brahmins who assisted at the offering” (Gowan to Boulderson, April 25, 1836; BL, IOR/V/27/68/233:452).^6^ Following an epidemic across Garhwal between 1834 and 1835, during which 633 people died, in 1836, Mahamari affected several villages in pargana Budhan, causing further 140 deaths.^7^ This led to the first direct observations of the disease on the part of British colonial officers, with Revenue Officers noting swellings under the armpits and knees, and death occurring in two to four days, as well as that the disease was curiously “preceded by a mortality among the rats in the village” (BL, IOR/V/27/68/233:451).^8^

To contemporary readers, swellings under the armpits and rats dying before humans leaves very little to the imagination, as the coexistence of these phenomena are strongly associated with the image of bubonic plague. This was not the case at the time, however, as regards British colonial and metropolitan audiences. On the one hand, swellings were seen by doctors as part of several other pathologies, and, on the other hand, before the end of the nineteenth century rats were not believed by either British scientific or lay audiences to be carriers or spreaders of plague or any other infectious disease (Pemberton 2014). If rats had long been considered to be detrimental to property and food resources, their only redeeming characteristic was widely held to be their supposed disease-free nature (Fissell 1999). Indeed, in mid-nineteenth century England, the rat was considered uniquely able “to ‘clean’ and preserve itself from contamination by the filth and miasma of the sewer” (Pemberton 2014:532). In his study of perceptions of rats at the time, Neil Pemberton (2014:533) notes that, “rather than being correlated with plague, the sewer rat’s appetite for putrefying matter saved human inhabitants from ‘periodical plagues.’”^9^

Faced with what appeared as a perplexing coincidence of phenomena, and a disease that seemed to be highly transmissible, Batten decided that it was urgent to establish once and for all the nature of Mahamari, and clarify whether it was true plague or not, as well as if it could be cured or prevented. On March 4, 1850, the Medical Board of Calcutta appointed the Superintending Surgeon of the Meerut Circle, Dr C. Renny, to investigate Mahamari (BL, IOR/V/27/68/233). The results of Renny’s expedition were relayed in a document titled “Notes for a Report on a disease prevailing in Garhwal, locally called Mahamuree or Great Plague” (August 19, 1850) (BL, IOR/V/27/68/233). Information contained in the report was gathered by means of site visits, the examination of two patients, as well as verbal inquiries conducted by public *chaprasis* under Renny’s instructions.^10^ Renny maintained that, of the evidence gathered, some were superior to others. In particular, he ranked indigenous knowledge at the bottom of the evidentiary scale: “care will be taken to separate the facts observed or resting upon what is considered good evidence, and other circumstances depending upon hearsay or less perfect proof, and upon the ideas and prejudices of the natives” (BL, IOR/V/27/68/233:415). “Among the latter,” Renny argued, “are many particulars that may be true, but they require elucidation; to one only is the present report decidedly opposed, namely, their fears of contagion” (BL, IOR/V/27/68/233:415). Given at the beginning of Renny’s report, this statement is of great importance. For, more than simply pointing at a generic colonial attitude that framed indigenous subjects as driven by “prejudice” or “superstition,” it forms the key for understanding the manner in which ethnographic data were evaluated epidemiologically in the context of the first Mahamari investigation.

Reflecting a much-broader anxiety about plague in the first half of the nineteenth century, one of Renny’s key concerns was to clarify whether Mahamari was not simply a disease afflicting its victims with inguinal and axillary swellings, but “pestis” or “the plague of Egypt;” in other words what at the time was generally held to be “true” or contagious plague (BL, IOR/V/27/68/233:417). Renny’s entire report revolved around the proof of the fallacy of this identity, and the proof, on the contrary, that Mahamari was “not contagious, and simply a typhus of a very malignant kind, most probably infectious at all times” (BL, IOR/V/27/68/233:417). Renny thereby maintained that Mahamari was closer to the so-called Pali Plague than to the “Egyptian plague.”

Breaking out in March 1836, the “Pali Plague” had mainly affected the Rajasthan cities of Pali and Jodhpur, reducing the population of the former by half. Colonial reactions to the disease involved the familiar debate regarding whether the disease was “true plague.” As Mark Harrison (1999) has shown, following the usual disease ontology at the time, this debate was tied to the question of whether the disease was contagious or not; in other words, whether it was caused by means of human-to-human transmission (directly or indirectly, through items contaminated by humans) rather than through “the ‘infectious, atmospheres said to arise from filth and rotting matter” (Harrison 2012:9). As was often the case, no consensus was reached on this subject by the end of the epidemic in 1838, or indeed later. Whereas sanitarians like James Ranken (1839) disputed Pali Plague’s contagious nature, in a manner consistent with associating anticontagionism to the economic interests of the particular group across the globe at the time (Harrison 2012), Frederick Forbes accused the refuters of contagion as being in the grip of the “mercantile community” and insisted that the disease was *pestis* (Forbes 1849:59).^11^ If Renny sided with the non-contagious identification of the Pali Plague, and in turn identified Mahamari as a similar disease, what troubled his disease ontology was the contradiction of his definition of Mahamari as non-contagious by the ethnographic data at hand. For the inhabitants of the affected region had been consistently recorded as being convinced of what in colonial medical eyes classified as the contagious nature of Mahamari, as they were said to believe that the disease was transmitted through domestic items, most commonly by means of ghee jars (BL, IOR/V/27/68/233). Such was the fear of Mahamari among the local population, that they evacuated their villages and fled to the hills not only upon observing fellow villagers being sick, but also at the first sight of ratfalls, which they claimed preceded human outbreaks of the disease.

First reported by the third Commissioner of Kumaon, Colonel G. E. Gowan, in his 1836 report on the disease, indigenous accounts of ratfalls preceding human cases elicited great interest in Renny, who noted that “no other animal has been observed to be affected in the same manner, or by the epidemic generally” (BL, IOR/V/27/68/233:427). Indeed he explained that so universal and confident was this belief among inhabitants of the region that he was led to investigate further. While he did not have the opportunity to observe the veracity of the claim himself, as during his expedition no new outbreak took place, he was still able to ascertain that, a month earlier, the inhabitants of Mason had evacuated their village, “not of the disease breaking out or any death within the place arising from it, but only of the usual fears caused by the death of the rats” (BL, IOR/V/27/68/233:427). Renny visited the village on May 10, 1850, and found it to be still deserted, with its inhabitants holding up in the surrounding hills where from they would descend in the daytime for harvesting, only to return back to the hills for refuge. This led him to speculate that, “this murrain may be caused by poisonous food, or by mephitic vapours, and, in want of all proof upon the former, the probability is in favour of the latter,” and that, “a search after these might lead to some discovery on the unknown agent of the production of Mahamurree itself” (BL, IOR/V/27/68/233:428).

In his expedition journal (entry dated May 9, 1850), Renny described in detail his visit to Dadoli. There, he found that the village had been deserted after the inhabitants of three houses fell ill with the disease, which had been proceeded, according to the locals, by a rat epizootic.^12^ He asked the sole adult survivor about this sequence of events: “He acknowledged he had seen four dead together in the huts, but did not know of more, and even this number all at once in a house might raise the belief of its depending on some unusual cause where means are not taken to destroy the rats” (BL, IOR/V/27/68/233:432). In another journal entry, dated May 17, 1850, Renny noted that in the village of Sarkote, a middle-aged chataee-mat-maker named Kooto revealed that, “the rats in the house first sickened and died, he says, threw up blood” (BL, IOR/V/27/68/233:438). However, Renny remained skeptical about the ethnographic evidence gathered in the course of his investigation. In his exposition of the information he collected about the August-December 1849 epidemic in the village of Muhroree, where sixty-one people had perished of Mahamari, he related that a man named Gubnoo, the only patient to have survived the disease, cast doubt as to whether rat deaths actually preceded human ones: “He distinctly says that *after* two children had first died, the rats were found dead six or eight in a house” (BL, IOR/V/27/68/233:435).

Rather than focusing on their epistemic capaciousness or their potential status as etiological indexes, the manner in which Renny interpreted and evaluated indigenous responses to and framings of Mahamari outbreaks in the region was doubly negative. On the one hand, for the British colonial doctor, abandoning the sick and fleeing to the hills was not only an erroneous manifestation of native contagionism, but also dangerous for public health. For it resulted in a number of abandoned human corpses rotting in the evacuated villages: “this exposure of the dead has been a chief cause of the disease being kept up in the country” (BL, IOR/V/27/68/233:439). This, in Renny’s mind, was because corpses formed a source of “pollution of the air, by which the virus of an epidemic is extended” – a common “infection”-focused etiology at the time (BL, IOR/V/27/68/233: 423).^13^ Here we need to note the system between “contagion” and “infection” created by Renny. Believing Mahamari to not be “true plague,” and hence to be non-contagious, the colonial doctor forged an image of indigenous understandings of Mahamari as contagious as leading to the creation of a new, significant source of “infection” (exposed corpses) that maintained the disease in the region, irrespective of its mysterious original source. Reasoning that, “the total neglect of the sick by their friends […] and the terror thereby impressed upon them, must no doubt hasten the catastrophe,” Renny thus urged that, “This terror of the people must be overcome, for the over-precaution is unnecessary, and communication might be maintained at a moderate distance” (BL, IOR/V/27/68/233:438). In true sanitarian spirit, what Renny proposed as a priority for stopping and preventing the “infection” was the removal of corpses from the vicinity of dwellings (BL, IOR/V/27/68/233:423).

Renny’s concern over the “epidemic corpse” (Lynteris and Evans 2018) forms part of a complex epidemiological anxiety over human cadavers, which defies dichotomous histories of miasmatic/germ-theory etiologies of disease. The epidemic corpse has been both an object of and an agent for epidemiological thinking at least since classical times, with narratives of corpse disposal inherited from Thucydides, Lucian, and Ovid becoming entangled with shifting etiological and public health frameworks for centuries in Europe and the Middle East (Gardner 2019). For mid-nineteenth century British doctors, the association of the London Plague of 1665 with the supposed opening of earlier plague pits formed a common reference that was further enhanced by what Faye Mary Getz (1991) has called the “gothic epidemiology” of the time. In British colonial contexts in particular, indigenous corpses and their disposal formed a subject of horror and fascination, perhaps best exemplified in the British obsession over Hindu cremation. As examined by David Arnold (2021), this configured Indian and particularly Hindu corpses in colonial eyes into indexes of Otherness as well as into sources of pestilence and disorder. Seen in this context, Renny’s reduction of a complex indigenous understanding of and response to Mahamari (of which we are only offered a glimpse, given the lack of indigenous accounts from the time) to a problem of “infection” and “disinfection” via hygienic corpse disposal marks a characteristically colonial move of epistemic enclosure. This move selected ethnographic data that already fit colonial epidemiological or public health frameworks, so as to reinforce the latter by means of an image of pestilential otherness. But what of ethnographic data, such indigenous observations of ratfalls preceding human illness, which did not directly fit Renny’s framework of disease etiology and epidemic blame? While the possibility of a telluric, miasmatic source of the disease affecting both rats and humans was maintained by Renny, this was not what captured his attention. It was flight as a result of sighting ratfalls that was, by contrast, epidemiologically valorized. This is because flight became associated with a supposedly unreasonable fear of contagion, which ended up creating a source of “infection:” exposed corpses.^14^ In other words, for Renny, it was indigenous *response* to ratfalls as a superstitious, contagion-focused behavior that led to a new source of “infection” rather than indigenous *knowledge* of ratfalls preceding Mahamari as a potential source of etiological evidence on the disease that formed the significant epidemiological datum.

## Francis and Pearson’s expedition

Renny’s report was well-received by his superiors, with the Medical Board of Calcutta praising his work and supporting his advice for sanitary intervention. The Board also sanctioned further investigation (BL, IOR/V/27/68/233). In the course of these new studies, ethnographic data surrounding Mahamari would be radically reinterpreted without, however, leading to a recognition of rats as a source of plague. In 1852 the Board decided that two Medical Officers should be stationed in a location where they could “travel frequently through the infected districts, to carry sanitary rules into effect, and simultaneously investigate the nature of the disease” (Francis and Pearson 1854:625). Behind the scientific goals of this decision lay a more pragmatic one, as expressed by the Lieutenant-Governor of the North-Western Provinces, who stressed the need “to give the people spirit and confidence by showing them, that the Government feels and sympathizes with their sufferings, and is prepared to make every possibly exertion for their relief” (Francis and Pearson 1854:625). The doctors selected for this task were Drs Charles Richard Francis and Frank Pearson, initially over a period of two years, with Pearson continuing for twenty-five years (to 1875) on a permanent basis as Sanitary Officer of Garhwal with duties including the supervision of Mahamari and its control (Francis 1880).^15^

Francis and Pearson arrived in the region in August 1852, during a major Mahamari outbreak. In the course of their first tour, they examined fifty villages “with the view of comparing these foul spots, both in health and in disease” (Francis and Pearson 1854:627). The doctors agreed that Mahamari was “true plague,” i.e. contagious, an idea supported by other medical experts in the region at the time, such as Civil Assistant Surgeon of Moradabad, W. S. Stiven (BL, IOR/V/27/859/20:6). In his *Report on the Epidemic in the Moradabad District in 1854*, Stiven contested the opinion that the disease was non-contagious, quoting Renny’s own remarks on the flight of locals at the first sight of the disease (BL, IOR/V/27/859/20). Rather than dismissing this as superstitious fear, Stiven explained, it should be taken as good evidence of the contagious nature of Mahamari. For, he reasoned, under normal circumstances, “a native, whether he be hill or plainsman, is affectionately fond of his family, and will remain with it, to defend and protect it (for instance in the case of fire) at the risk of his life” (BL, IOR/V/27/859/20:15). In an argument that was much more attuned to indigenous ethics of care than Renny’s, Stiven argued that the radical decision to break with established mores and take flight was driven by “a practical conviction that the disease is poisonous to the touch […] in other words, that it is contagious” (BL, IOR/V/27/859/20:15); a sound understanding of the nature of the disease, in his opinion, that led locals to break with their moral and religious code so as to save their lives.

Francis and Pearson (1854:628) also took indigenous reports of ratfalls seriously, noting: “how sure an index was the death of rats to approaching ‘Muhamurree.’” That failing to flee before the sight of ratfalls was fatal was exemplified in the case of the village of Malsee: “when we visited the village the first time, the inhabitants were in great trouble. The rats were dying, and they knew they ought to go; but home was home, and they lingered still. On our second visit, a week afterwards, Mahamuree had broken out amongst them” (Francis and Pearson 1854:628).

So impressed were Francis and Pearson by these observations, that they proceeded to dissect a rat, performing what in all probability is the first autopsy of a rat in the context of a plague-related investigation in history. This showed that the lungs of the animal were punctuated by “several black carbonaceous-looking patches” (Francis and Pearson 1854:634). The lung pathology of the dissected rat was particularly significant, as it rhymed with human pathology observed in ten cases contained in the doctors’ report, as well as with their theory that Mahamari was a blood disease originating in the lungs where “congestion supervenes upon inspiration of the poison” (Francis and Pearson 1854:635).

According to Francis and Pearson, plague was the sole disease where “the death of animals is coincident with that of human beings” (Francis and Pearson 1854:638). What made the rat special, in their opinion, was its association with “filth” – the universally accepted source of plague at the time – and its habit of living and thriving in “the foulest locations” such as city sewers: “Here filth of every description is accumulated, and emanations from human dung, slaughtered animals, &c., &c., poison the whole surrounding atmosphere” (Francis and Pearson 1854:638). Following current precepts regarding the history of miasma theory, we may be inclined here to assume that by this description the colonial doctors were drawing an identity between filth and rats, or that they claimed that, as living personifications or carriers of filth, rats spread the miasmatic agent (often called a “poison”) that caused plague. Instead a very different operation in epidemiological reasoning was in fact in place; one that placed emphasis on the exceptionality of local conditions, and which confirms Prashant Kidambi’s (2004) broader analysis of British colonial approaches to plague as an “infection of locality.” For, Francis and Pearson reasoned, if thousands of rats managed to “breed and thrive” in as filthy places as the London and Paris sewers, “It is evident, therefore, that the so-called poison of ‘Mahamurree’ must be a peculiarly virulent one, to destroy a class of animals which select the foulest atmosphere in nature for their abode” (Francis and Pearson 1854:638).

What we have here then is neither a “pythogenetic” continuity between rats and plague, nor a framing of the rat within miasmatic or more generally “infection”-focused understandings of the generation of pathogenic “animal poisons” (Lynteris 2019).^16^ Instead, what rats dying of Mahamari proved to Francis and Pearson was the extreme virulence of the disease in the specific location, which led even such animals as habituated to the “foulest atmosphere” to succumb to Mahamari. Thus ratfalls became an index of the exceptional pestigenic conditions prevailing in the affected locality. This reflected and in turn enhanced Francis and Pearson’s broader interpretation of Mahamari as a disease tied to specific local conditions: like a familiar English shrub can be found growing in the wild in the Himalayas, they reasoned, if “the conditions for its development are fulfilled,” so with Mahamari, which cannot be “generated in the well-ventilated dwellings of European society” any more than “the acorn will yield an oak in the plains of Hindoostan” (Francis and Pearson 1854:639). An exceptional ability to produce plague was attributed not to the natural environment of the region (its climate, latitude, soil, etc.), but to the living conditions of indigenous dwellings and to insalubrity resulting from herding and agricultural activities around them (particularly, dung heaps and manure pits).

The contrast between the “purity of the natural atmosphere” in the region and the “state in which the villages are kept” was drawn time and again in colonial reports on the region (e.g. BL, IOR/V/27/68/233:46; Ramsay and Buck 1874). For Francis and Pearson, the interior of Kumaoni and Garhwali dwellings was characterized by an extreme “vitiation of the air;” so much so that indigenous houses were compared to “a brewer’s vat” saturated by carbonic acid – a miniature of the Black Hole of Calcutta or the Neapolitan Grotta del Cane, where CO_2_ rendered the blood of the inhabitants “vitiated” in turn (Francis and Pearson 1854:639, 640).^17^ The metonymic operation in place is notable. On the one hand, the two doctors mobilized the image of the natural carbon-dioxide emissions taking place inside the cave known as Grotta del Cane (cave of dogs) in Foro di Pozzuoli near Naples, in Italy. These formed part of a popular mid-century spectacle (often included in the so-called “Grand Tour”; Fleming 2016) that involved tourists watching dogs being introduced into the cave where they would suffocate and perish as a result of exposure to carbonic gases (Swaine Taylor 1832). On the other hand, the image of Kumaoni and Garhwali houses as miniatures of the Black Hole of Calcutta evoked one of the most powerful tropes in British colonial discourse: the narrow guardroom in Fort William where British and other European soldiers had been imprisoned for three days in 1756 by the Nawab of Bengal, and where, according to the now contested colonial narrative, the majority of them perished as a result of suffocation, attributed to an excess of CO_2_ or the lack of oxygen (Chatterjee 2012).

Francis and Pearson’s depiction of indigenous houses as miniatures of both the Neapolitan Grotta del Cane and the Black Hole of Calcutta first animalized the inhabitants of these infrastructures, evoking an image of them as dogs, and then legitimated colonial intervention as a force of liberation and progress by portraying locals as captives in Black Holes of their own making, who moreover threatened those around them by allowing the emergence of a disease as contagious and lethal as plague. The use of the “miniature” trope in this colonial context thus came to generate what Emily Senior (2018:194) has called a “medicalized aesthetic sensorium” that forged together two phenomena famously associated with air vitiation at the time, the one natural (Grotta del Cane) and the other artificial (Black Hole), in a single and highly-evocative epidemiological image of an indigenous way of life as the source of pestigenic air-vitiation.

## Shifting colonial medical approaches

Spanning a period between 1836 and 1860, the reports and other documents on Mahamari that I have examined in this article indicate that colonial medical understandings of the disease at the time conformed with what Mark Harrison has identified as a shift in British epidemiological thinking in India toward a framework dominated by ideas about filth (Harrison 1999). Moreover, reflecting the chronology of British epidemiological thought drawn by Michael Brown (2008b:527), by the mid-century, problematizations of Mahamari as a disease of filth depended on ideas of vitiated air, not as “a *general* [i.e. climatic] constitution” but as a “*specific* corruption of the atmosphere” tied to local infrastructures and practices. However, in the case of British colonial doctors in charge of the explanation and prevention of Mahamari, the embracement of an etiology based on filth and vitiated air was not concurrent with anticontagionism. For, disputing Renny’s report, subsequent works and policies accepted the disease as being highly contagious. In this contagion-infection hybrid epistemological context, ratfalls functioned as indexes not of the filth-producing properties of rats, or of some ability of the animal to generate infectious miasma or animal poison, but of the extreme, anthropogenic vitiation of the air in the locality.

Whereas Renny had overlooked indigenous knowledge of ratfalls as a potential source of epidemiological information so as to focus on indigenous response to them as an irrational and pathogenic behavior, Francis and Pearson valorized indigenous knowledge, but only so as to further pathologize indigenous lifeways. Kumaoni and Garhwali accounts of ratfalls were taken seriously by the two doctors, leading them to seek a scientific explanation of why rats died of a disease that was widely held to not affect them. The explanation arrived at by Francis and Pearson indicted local modes of habitation as leading to an unprecedented virulence of the plague “poison,” so much so that *even rats* succumbed to it. Here we have a different operation of epidemiological reasoning with ethnographic data than that encountered in Renny’s investigation. Both indigenous knowledge of and response to ratfalls are taken seriously and seen as correct and reasonable. What is indicated as the cause of the ongoing epidemic is no longer Kumaoni or Garhwali superstition and misreaction to outbreaks, but the underlying conditions of indigenous lifeways.

The critical shift in epidemiological reasoning and epidemic blame is double. On the one hand, it is a shift in the valorization of ethnographic evidence: dismissed by Renny, and taken seriously by Francis and Pearson. On the one hand, it is a shift in the etiological scrutinization of indigenous lifeways: for Renny the cause of recurring outbreaks is a mistaken belief (in Mahamari as a contagious disease) and its subsequent “superstitious” and “infection”-producing reaction (fleeing and abandoning pestilential corpses), whereas for Francis and Pearson it is the way Kumaoni and Garhwali people built and inhabit their houses.

The shift encountered here is one that transfers epidemic blame from an isolatable belief to a much broader range of indigenous lifeways, including housing infrastructures and ways of inhabiting them. What was the practical result of this? The immediate measures taken involved a most forceful intervention on indigenous lifeways that affected key aspects of what following Tim Ingold (2000) we could call Kumaoni and Garhwali “dwelling,” for this was portrayed and acted upon as what we may call a “dwelling for plague”. “One month was given to the people to turn all the cattle out of their villages, to sweep, white-wash and ventilate; in fact to convert their homes into an approach to something civilized,” wrote Pearson, stressing that when such measures were not undertaken, “Four or five startling *fines*, however, electrified them into instant obedience, and before another month had elapsed, to use the people’s own expression, you might have eaten your dinner off the very stones of the village. Mahamurree had now no dwelling place, and had departed” (BL, IOR/V/23/121, Pt 35 Art 4:^1861^, emphasis in the original). Evacuated and sanitized houses had to remain empty for three or four months, without specifying where their occupants lived during that period. Indeed, Peason himself confessed that, before deciding that such measures sufficed to stop outbreaks, he “was in the habit of burning down all infected houses” (BL, IOR/V/23/121, Pt 35 Art 4:27).

Pearson would remain Sanitary Officer of Garhwal until 1875 (House of Commons Papers Cd.140). Following a resurgence of Mahamari in 1860 (1000 casualties), which he attributed to the neglect of sanitation following the 1857 Indian Rebellion, he composed a new report, which brings our story to a close. In it, he described Garhwali houses in some detail, stressing their lack of ventilation and how the co-habitation with animals rendered the “floor well saturated with urine and dung” (BL, IOR/V/23/121, Pt 35 Art 4:1861:25). Moreover, Pearson described Garhwali dwelling habits in the bleakest manner, stressing that this was not due to poverty but to “habit and choice” (BL, IOR/V/23/121, Pt 35 Art 4:26). The racialized narrative that forms the basis of the etiological account of Mahamari in Pearson’s 1861 report reaches its nadir in a radical revision of the question of rats. For while Pearson still praised fleeing villages upon encountering the first Mahamari death as essential for survival, he argued that, in spite of being aware that ratfalls proceeded human deaths, Garhwali people failed to flee their villages upon encountering dead rats: “If they would but take the warning of the disease that is always sent them, viz., rats dying and dead all over the village, and fly at once, they might escape the pestilence altogether; but they wait until one of themselves sickens or dies, and not until then will they go. They are a strange race; they know the warning as a sure precursor, and yet they remain” (BL, IOR/V/23/121, Pt 35 Art 4: 26). Pearson’s conclusion thus marked a third move in the colonial enclosure of indigenous knowledge, where the supposed failure of Garhwali people to act according to their knowledge of Mahamari was used to foster a racialized portrayal of the former as fundamentally lacking in reason, and as too attached to bonds of kinship or norms of caring, even if he acknowledged that they possessed a valuable and correct epidemiological knowledge.^18^ It is tempting to see Pearson’s 1861 enclosure of indigenous knowledge as a mirror-image of that attempted by Renny twelve years earlier. If Renny castigated *fleeing* as a pathogenic reaction to Mahamari based on a superstitious approach to the disease, Pearson blamed *non-fleeing* as a failure to act reasonably, or according to one’s correct knowledge of Mahamari, and thus prevent human infection.

## Conclusion

If Rohan Deb Roy (2018:271) is right in arguing that “Empire […] could neither precede nor outlive nor be delineated from the discourses about science and nonhumans that it occasioned,” it is important to expand this condition to the inclusion of colonial discourses and corresponding policies on indigenous knowledge about nonhumans, including infectious diseases. While colonial approaches to Kumaoni and Garhwali understandings of Mahamari involved processes of “subordinating and recasting Indian knowledge” (Bayly 1996:224), the forms assumed by different colonial enclosures of indigenous knowledge were not based on pre-determined colonial understandings of Mahamari, but were part of a dynamic negotiation of the nature of the disease and the measures needed to contain and prevent it. In Deb Roy’s terms (Deb Roy 2018), we can say that, as colonial medical categories, Mahamari and indigenous knowledge of Mahamari were continually *co-constituted*; a process that would continue after the timeframe examined in this article and into the bacteriological age, during the third plague pandemic, when Mahamari would become entangled with broader imperial scientific projects on plague, its origins and indigenous knowledge thereof across the globe.

In this article I have shown how colonial configurations of indigenous knowledge of Mahamari shifted between 1849 and 1861. For Renny (1849) what he considered to be the superstitious indigenous fear of the disease as contagious led to the abandonment of human corpses in villages, which came to function as persistent sources of Mahamari “infection.” For Francis and Pearson (1852–53) fleeing villages was correct and justified, and indigenous observations of ratfalls pointed at the existence of an extremely virulent plague “poison” in the locality, caused by the way in which Kumaonis and Garhwalis built and inhabited their houses. Finally, for Pearson (1861) fleeing was still correct and justified, but Garhwalis actually failed to flee upon sighting ratfalls, though they knew these preceded human cases. At the two extremes of these configurations stand Renny’s and Pearson’s enclosures of indigenous knowledge, which form racialized mirror-images of one another. The mid-ground was as it were, held by Francis and Pearson’s mid-1850s report, when indigenous agents were recognized for the first time as both knowing and responding to Mahamari outbreaks correctly. Still the concrete impact of this recognition was the adoption and implementation of public health measures that directly and violently impacted indigenous lifeways, fostering a framework of “intrusive intervention” (Mooney 2015) that, while not denigrating indigenous rationality, pathologized, proscribed and transformed the material conditions of Kumaoni and Garhwali life, its modes of habitation, infrastructures and interspecies proximities.

The examination of this neglected historical case of colonial medical investigation into the nature of a contested disease in the Indian Himalayas underlines the importance of placing more emphasis on the practical impact of colonial epistemic enclosures of indigenous knowledge in relation to animal and zoonotic diseases. Approaching colonial medical epistemologies as practices that relied on a dynamic interplay with indigenous knowledges and which, at the same time, had an impact on indigenous lifeways that was in some cases more severe when the latter were credited rather than dismissed is necessary if we are to understand the epistemic-political entanglement involved in the institution of infectious disease and epidemic control frameworks past and present.

## Notes

1. The term Mahamari literally means “the great disease” and was used in other contexts in India to refer to epidemic diseases other than plague – I would like to thank Abhijit Sarkar for this information. However in the Kumaon and Garhwal literature “Mahamari” always and unambiguously refers exclusively to a disease believed by colonial doctors to be bubonic plague.

2. In this article, I do not attempt to recover Kumaoni and Garhwali knowledge of Mahamari, nor to reconstitute indigenous interspecies relations as these played out in the mid nineteenth century, as such information is unfortunately not available in the archives I have been able to access. Nor do I attempt to reconstitute a history of Mahamari plague; something that would require its examination into the late nineteenth and early twentieth century, when, during the third plague pandemic (1894–1959), the bacteriological identification of Mahamari as plague led to a co-option of earlier studies of the disease into a narrative of discovery of the zoonotic mechanism of plague transmission (see for example: Chaytor-White 1902). For a discussion of the medical research on and public health approaches to rats during the third plague pandemic in India see: Silva (2020), Evans (2018), Mukharji (2017). For a general history of the first years of the third plague pandemic see Echenberg (2007).

3. Relying on my earlier work on plague and marmot-hunting in Mongolia (Lynteris 2015, 2016), as well as on the wider literature on knowledge enclosure (e.g. Federici 2009; Fisher 2022; Joranson 2008), by the “enclosure of indigenous knowledge” in this article I mean the process through which colonial interpretations and representations of indigenous ways of knowing dispossessed the latter of their epistemic autonomy by making them understandable only in relation to western, colonial systems of knowledge.

4. The population of British Garhwal in 1852 was 132,774 individuals (BL, IOR/V/27/314/695).

5. A manuscript copy of reports, correspondence and other documents on Mahamari appearing in BL, IOR/V/27/68/233 are contained without variation in: BL, IOR/F/4/2472/138452. With the exception of “Garhwal” and “Kumaon,” whose spelling is kept constant to avoid confusion, in this article I have maintained the spelling of locations as it appears in the archives examined.

6. Built at an altitude of 3,583 m, the Shaivite temple of Kedarnath has been an important pilgrimage site along the Chota Char Dham circuit. The rawal is the chief priest of the temple. Sashters are canonical interpretations of the Vedas.

7. A pargana (also spelled *parganah*) was an administrative unit.

8. Later the rats were identified as “Norwegian rats” (House of Commons Papers, Cd.140).

9. The quotes in Pemberton are from: Rodwell (1858).

10. Renny dismissed information about Mahamari in previous published sources as not based on direct observation (BL, IOR/V/27/68/233). Chaprasis were junior official messengers.

11. For a discussion of Ranken, Forbes and anticontagionism, see Harrison (1999:197–98, 202–203). Forbes (1849:34) also mentioned ratfalls in the village of Tai’wali, during the latter half of April, and “just before its first appearance,” but attributed no significance to this datum, which simply rhymed with observations of broader epizootics among cattle and poultry; a leitmotif of European descriptions of epidemics since Ovid (Gardner 2019).

12. Renny did not use the term “epizootic” or “ratfall” (the current terms for the phenomenon in scientific literature), but “murrain” instead (BL, IOR/V/27/68/233:428).

13. The meaning of “infection” in the mid-nineteenth century remains a topic of intense historical debate. Barnes (2014) has provided an excellent new approach to the subject, which, while maintaining the importance of atmospheric framings of “infection” at the time, also stresses the need to recognize a parallel system of “infection” as something supposedly spontaneously produced by certain objects or materials.

14. On contagionism as superstition see: Cooter (2013). On superstition as a source of contagion see: Senior (2018).

15. The doctors’ expedition was also credited with introducing vaccination against smallpox among Bothiya trans-Himalayan trader groups in the region (Anon. 1868).

16. On pythogenesis see: Kidambi (2004) and Naraindas (1996).

17. On mid-century ideas about vitiated air and its relation to epidemics see: Carter (1981), Barnes (2006) and Kiechle (2017).

18. Cf the case of plague in Inner Asia, where Mongols and Buryats, “though praised for their knowledge and for containing plague” were relegated “to a static ontological condition: that of functional unreason” Lynteris (2016:160–161).
